# Extensively Drug-Resistant Tuberculosis in the Time of COVID-19—How has the Landscape Changed for Pakistan?

**DOI:** 10.1017/dmp.2020.230

**Published:** 2020-07-07

**Authors:** Mahnoor Islam

**Affiliations:** Dow University of Health Sciences, Karachi, Pakistan

**Keywords:** COVID-19, tuberculosis, XDR-TB

## Abstract

The incidence of extensively drug-resistant tuberculosis (XDR-TB) has been rising consistently in Pakistan, and the country is likely to experience another surge of cases in the midst of the COVID-19 crisis. It is imperative to consider how the rising proportion of XDR-TB is best tackled during the pandemic; this includes finding a solution to the problem of non-adherence at the level of community-based healthcare, the utility and practicality of simultaneous testing for COVID-19 and TB, and reconciliation of the World Health Organization’s recommendation of home-based treatment with the need for frequent monitoring of anti-tubercular therapy in XDR-TB. Operational research is needed expeditiously to bypass these limitations.

Tuberculosis (TB) has established itself as the world’s leading infectious-disease killer, causing approximately 1.5 million deaths annually around the globe. Notably, a disproportionate 95% of these deaths are restricted to middle-income/low-income countries, one of which is Pakistan. Ongoing efforts to control the spread of TB, although consistent, have been hindered by a lack of patient awareness, unemployment or underpaid labor resulting in financial constraints, and the social stigma of tuberculosis itself, all of which collectively discourage patients from adhering to treatment.^1^ This inadequate/incomplete treatment is thought to give rise to extensively drug-resistant forms of tuberculosis (XDR-TB), the incidence of which has been periodically rising each year.^2^

A recent report by the Stop TB Partnership suggested that efforts against TB may be set back even further during the coronavirus disease 2019 (COVID-19) crisis, with 6.3 million new cases and 1.4 million deaths expected worldwide between now and 2025^3^; for Pakistan, it is imperative to consider how the rising proportion of XDR-TB may require a unique approach to this situation. For one, the stigma of respiratory symptoms faced by patients with TB is now compounded by the added threat of COVID-19, making presentation/follow-up to healthcare professionals even less likely and providing opportunity for drug resistance to take root in the gaps of incomplete therapy.

At the institutional level, the information note by the World Health Organization (WHO) has raised certain concerns regarding how healthcare facilities may alter their approach to TB in the COVID-19 crisis.^4^ The fifth recommendation of the note addresses the utility of simultaneous testing for both conditions, wherein WHO suggests an individualized patient assessment based on prognostic factors, history of exposure, and local burden of disease. Generally, investigating TB in COVID-19 patients after a week of observation for alarming symptoms (or earlier, for patients with co-morbidities) is reasonable; however, in Pakistan, where national TB burden is high and rising despite control efforts, ruling out concomitant TB in patients confirmed to have COVID-19 may be doubly important to prevent TB transmission to other patients in COVID-19 isolation wards. The questions arises, would it be advisable to immediately test for TB (including latent) even in patients without co-morbidities, if they report risk factors?

Second, WHO recommends home-based TB treatment during this time; however, the treatment of XDR-TB is often associated with considerable toxicity and requires frequent evaluations over several months. In these patients, it is imperative to develop home-based models of surveillance to ensure adherence and safety. While Web-based methods have been suggested for this purpose, they may not be entirely practical in low socioeconomic areas of Pakistan (which contribute the most to TB prevalence) due to a scarcity of digital resources.

To combat these problems; community-based health care must be optimized, with emphasis on structured counseling and family support. Operational research is needed expeditiously to find ways to bypass limitations. Furthermore, government-level action to prevent export bans and stockpiling may help to maintain a continuous flow of healthcare resources, especially with the aid of international partners for resource generation.
